# Effectiveness of acetylcholinesterase inhibitors and memantine in patients affected by severe dementia

**DOI:** 10.1007/s11357-025-02052-3

**Published:** 2025-12-15

**Authors:** Giovanni Zuliani, Gloria Brombo, Tommaso Romagnoli, Michele Polastri, Francesco di Paola Dario, Marco Zuin

**Affiliations:** Department of Translational Medicine and for Romagna, University of Ferrara, Via Savonarola 9, 44121 Ferrara, Italy

**Keywords:** Acetylcholinesterase inhibitors, Memantine, Dementia, Alzheimer’s disease, Cognitive decline

## Abstract

The efficacy of acetylcholinesterase inhibitors (AChEI) and memantine (ME) in severe dementia remains uncertain, and real world data are limited. We evaluated the progression of cognitive decline and mortality rates in a cohort of outpatients with severe dementia treated with AChEI and/or ME or not treated, from the National Alzheimer’s Coordinating Center Uniform Data Set (NACC-UDS). Overall, 703 patients (mean age:78.3 ± 7.2 years; females:56.1%) with severe dementia (Mini-Mental State Examination—MMSE ≤ 10/30) were included [407 late-onset Alzheimer’s disease (LOAD), 126 Lewy body dementia (LBD), 170 vascular dementia (VD)]. Patients were grouped by treatment status: untreated (AChEI − /ME − ; n.70), AChEI only (AChEI + ; n.294), ME only (ME + ; n.215), combined therapy (AChEI + /ME + ; n.124). During follow-up (mean:1.7 ± 0.3 years; range:0.2–2.9 years) the mean MMSE scores declined by − 1.7 points (Standard Deviation: -1.55 to -1.9) in AChEI − /ME − (p for trend:0.001), − 1.5 points (-1.55 to -1.9) in AChEI + (*p*:0.001), − 0.7 points (-0.5 to -0.9) in ME + (*p*:0.09), while it increased by + 0.7 points (+ 0.4 to + 1.0) in AChEI + /ME + (*p*:0.001). Survival rates were 8% in AChEI − /ME − , 10% in AChEI + , 28% in ME + , and 49% in AChEI + /ME + . In Cox proportional hazards analysis (age and sex adjusted), AChEI + /ME + exhibited a 39% reduction in total mortality compared with no treatment (HR:0.61; 95%CI:0.49–0.77; *p* < 0.001), ME + displayed a 22% reduction (HR:0.78; 95%CI:0.65–0.94; *p*:0.007), while AChEI + showed no association (HR:0.97; 95%CI:0.80–1.18; *p*:0.75). When the Cox proportional hazards models were stratified by dementia subtype, consistent significant patterns were observed in LOAD and LBD. In this real-world cohort of older adults with severe dementia, the combination therapy (ME + AchEI) seems to be associated with the best outcomes, including a stabilization of cognitive function and reduced total mortality.

**Graphical Abstract:**

**Figure:**
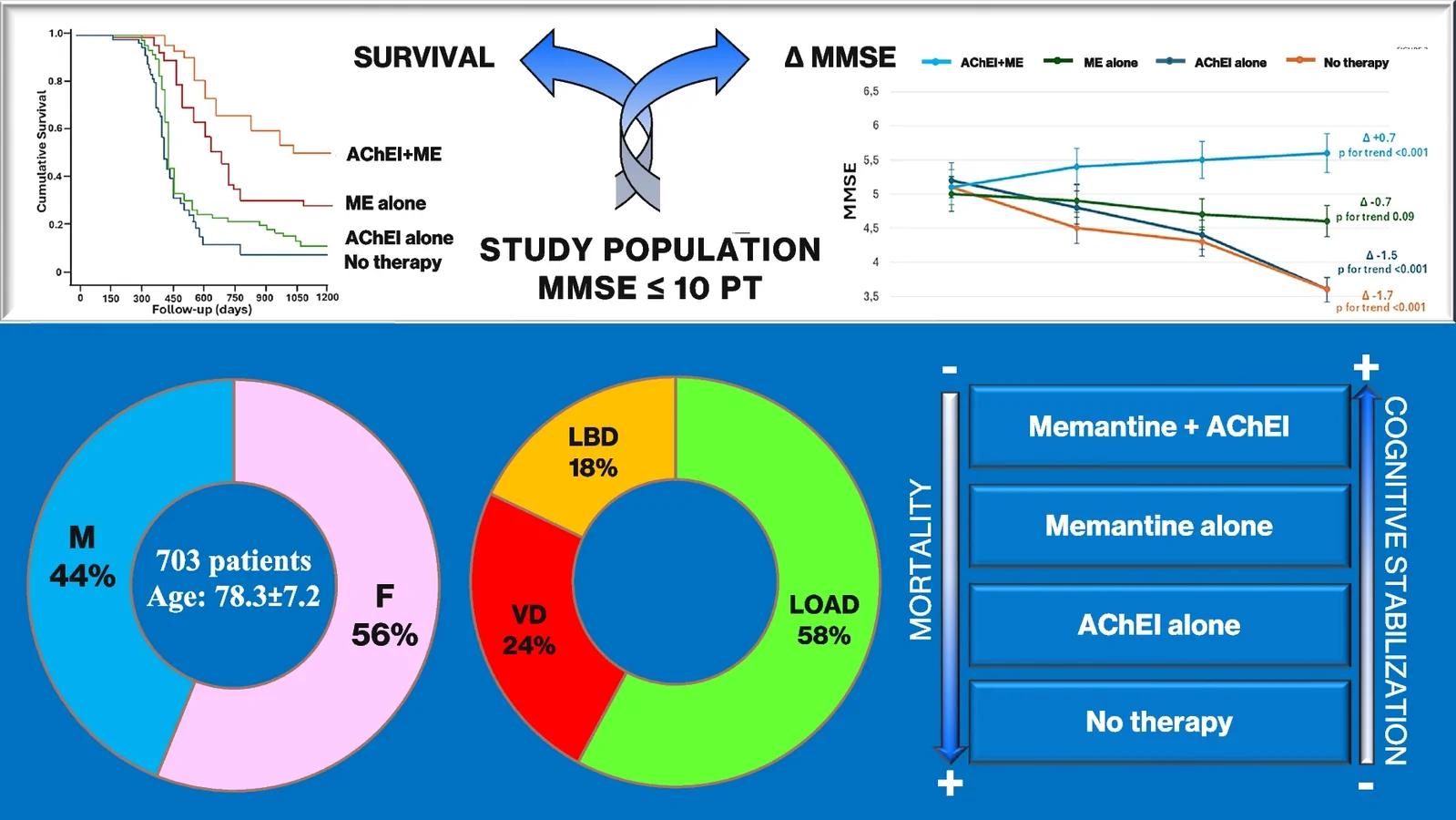

**Supplementary Information:**

The online version contains supplementary material available at https://doi.org/10.1007/s11357-025-02052-3.

## Introduction

Acetylcholinesterase inhibitors (AChEI) are the first-line pharmacological treatment for Alzheimer’s disease (AD) [1]. These agents inhibit cholinesterase enzymes, thereby preventing the breakdown of acetylcholine, increase its availability, and prolong its action at synaptic sites. Evidence from randomized clinical trials (RTCs) and post-marketing studies suggest that AChEI offer moderate clinical benefits in AD [1–4] and may also reduce the total mortality rate [4, 5]. AChEI can be prescribed also to treat Lewy body disease (LBD) [6, 7] and some studies have demonstrated their effectiveness also in vascular dementia (VD) [8]. For patients with severe AD defined by Mini-Mental State Examination – MMSE [9] score ≤ 10/30, AChEI use is authorized in some countries (e.g., the United States and Canada) but not in others (e.g., Sweden and Italy), due to uncertainties regarding their clinical efficacy in this population. Limited evidence suggests that discontinuing AChEI in patients with advanced dementia may lead to a deterioration in cognitive and functional outcomes [10]. Moreover, small RCTs from the early 2000s indicated that AChEI might still offer short-term benefits in severe AD [11–14]. Memantine (ME), an NMDA receptor antagonist, represents the second-line treatment for AD; indeed, over activation of NMDA receptors has been implicated in excitotoxicity, neuronal damage, and disease progression in AD [15]. ME has been shown to be effective in moderate-to-severe AD [16, 17] and is recommended for patients with MMSE scores ≤ 20/30, either as monotherapy or in combination with AChEI. Off-label, ME has also demonstrated some efficacy also in LBD and VD [15, 18, 19]. In patients with severe dementia, both AChEI and ME may be considered, but treatment decisions should be individualized, taking into account that: 1. MMSE may not fully capture cognitive and functional status at advanced disease stages; 2. drug tolerability can be a limiting factor due to potential side effects; 3. most evidence on combined AChEI + ME therapy comes from short-term trials, often yielding conflicting results, and conducted in mixed populations with moderate-to-severe AD [20–23].

In the present study, we evaluated the progression of cognitive decline (MMSE scores) and overall mortality in a cohort of outpatients with severe dementia (AD, LBD, or VD) enrolled in the National Alzheimer’s Coordinating Center Uniform Data Set (NACC-UDS), and treated with AChEI and/or ME or left untreated.

## Methods

Data were obtained from the NACC-UDS, a nationwide longitudinal database comprising standardized clinical data from approximately 34 current or former Alzheimer’s Disease Research Centers (ADRCs) funded by the U.S. National Institute on Aging (NIA) (naccdata.org).

In the present study, we analyzed data collected between 2005 and June 2020.

Patients were included if they met the following criteria: (i) age ≥ 65 years at baseline; (ii) clinical diagnosis of dementia, including late onset AD (LOAD), VD or LBD; (iii) completion of a baseline evaluation and at least two subsequent visits in-person, each including a MMSE, with a minimum follow-up duration of two months.

Conversely, patients were excluded if they had at least one of the following: (i) fewer than three in person-visits and/or relative MMSE consecutive evaluations and/or (ii) MMSE score ≥ 11/30.

Comorbidities and functional status were assessed at baseline by the medical team of the clinic by means of medical history, medical records, medical examination, and blood chemistry tests.

Local ethics committees at each of the sites approved the study, and all participants provided written informed consent. All procedures adhered to relevant ethical standards and regulatory guidelines.

### Dementia definitions

Dementia was defined as meeting criteria for AD [24] or VD or LBD [25, 26] defined as: 1) objective cognitive impairment (i.e. performances falling greater than 1.5 standard deviations outside the age-adjusted normative mean) in at least two cognitive systems (memory, language, attention or executive functioning), and 2: cognitive impairment contributes directly to impaired activities of daily living. The patients were categorized into 4 groups: 1. Those receiving no drugs (AChEI-/ME-); 2. Those receiving AChEI only (AChEI +); 3. Those receiving ME only (ME +); 4. Those receiving both the drugs (AChEI +/ME +). The AChEI and ME exposure was assumed to be constant over time.

### Cognitive decline evaluation

The MMSE was administered during the outpatient visits to evaluate the rate of cognitive decline over time [9]. The MMSE evaluates orientation, short-term and delayed recall, calculations, language interpretation, naming, and praxis; higher scores represent a better cognitive performance. It represents the most used cognitive assessment tool in dementia [27].

### Statistical analysis

The characteristics of the four groups of patients were compared using one-way ANOVA, the chi-2 test and the Kruskal Wallis test for the variables that were not normally distributed.

Difference in cognitive trajectories over time (MMSE score change) in the patients belonging to the four groups (AChEI-/ME-, AChEI +, ME +, and AChEI +/ME +) were estimated using mixed-effects repeated measures models of unstructured-variance–covariance matrix.

To assess the association between dementia pharmacotherapy and overall survival, we fitted multivariate Cox proportional hazards models to estimate hazard ratios (HRs) and 95% confidence intervals (CIs) for all-cause mortality across the four treatment groups (AChEI −/ME −, AChEI +, ME +, and AChEI +/ME +), using the untreated group (AChEI −/ME −) as the reference category. Models were adjusted for age and sex, and the proportional hazards assumption was verified through both visual inspection of log–log survival plots and statistical testing of covariate-by-time interactions. Additional stratified analyses were conducted within dementia subtypes (LOAD, LBD, and VD). Analyses were performed by SPSS for Windows statistical package, version 21.

## Results

### Patients’ selection and general characteristics

**Fig. 1:**
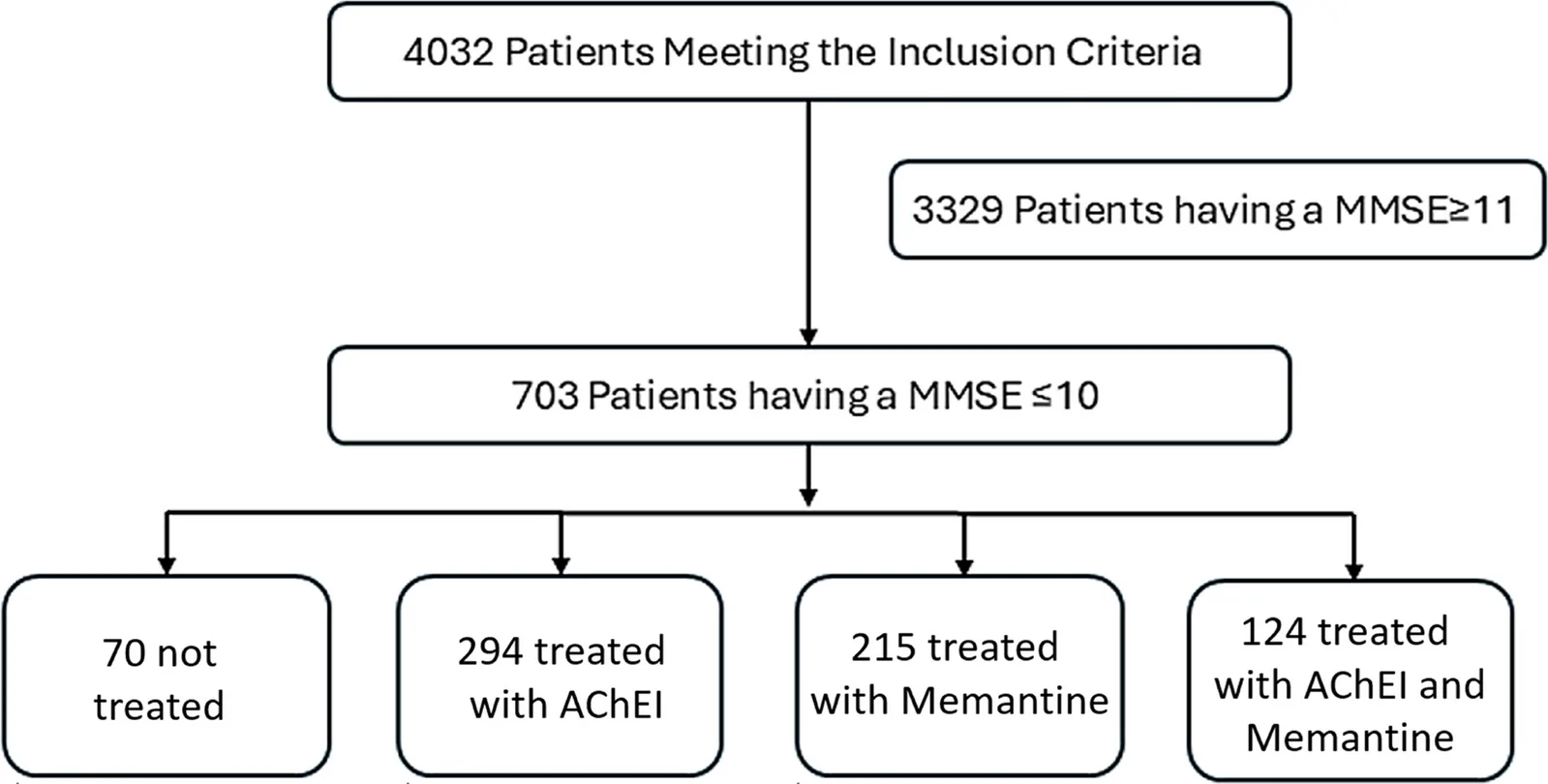
Study flow-chart

Of the initial 4,032 patients who met the general inclusion criteria, 3,329 were excluded due to having a baseline MMSE score ≥ 11/30. The remaining 703 patients constituted the final study sample (mean age: 78.3 ± 7.2, 56.1% females) (Fig. 1). Among them, 407 had LOAD, 126 LBD and 170 VD, respectively. At baseline, patients were categorized into four treatment groups: 70 were untreated (AChEI −/ME −), 294 were receiving AChEI only (AChEI +), 215 were treated with ME only (ME +), and 124 were receiving both AChEI and ME (AChEI +/ME +) (Table 1). The mean duration of follow-up was 1.7 ± 0.3 years (range: 0.2–2.9 years). There were no statistically significant differences between treatment groups in terms of comorbidities or concomitant medication use; the most prevalent chronic conditions were hypertension, heart failure, and coronary artery disease (Supplementary Table 1).

**Table 1:** Principal characteristics of the sample according to the treatment with AChEI/ME

	AChEI-/ME- *N* = 70	AChEI + *N* = 294	ME + *N* = 215	AChEI +/ME + *N* = 124	*p*
Age (years)	78.9 ± 7.4	78.4 ± 7.4	77.8 ± 8.1	78.1 ± 6.9	0.63
Females, *n* (%)	39 (55.7)	168 (57.1)	119 (55.3)	69 (55.6)	0.68
Years of education	13.6 ± 3.9	13.3 ± 3.4	13.7 ± 2.9	12.9 ± 4.4	0.53
Type of Dementia					
LOAD, *n* (%)	39 (55.7)	181 (61.5)	114 (53.0)	73 (58.8)	0.41
LBD, *n* (%)	13 (18.5)	52 (17.6)	40 (18.6)	21 (16.9)	0.63
VD, *n* (%)	18 (25.7)	61 (20.7)	61 (28.3)	30 (24.1)	0.51
*AChEI* acetylcholinesterase inhibitors, *ME* memantine, *LOAD* late onset Alzheimer’s disease, *LBD* Lewy body disease, *VD* vascular dementia					

### AChEI/Memantine treatment and cognitive decline

**Fig. 2:**
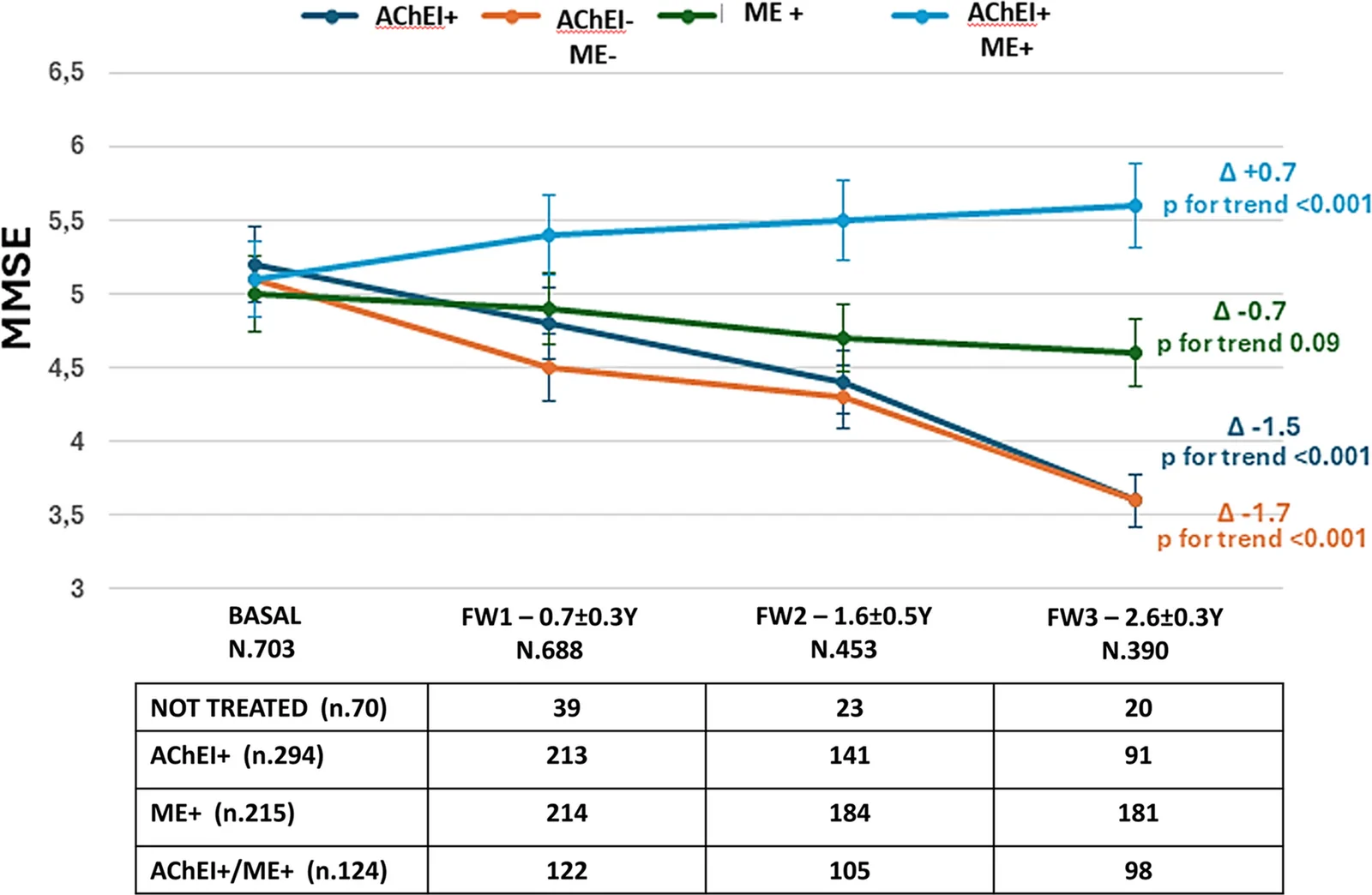
MMSE scores (mean ± SD) at baseline (n.703) and at follow-up 1 (0.7 years; n.688), follow-up 2 (1.6 years; n.453), and follow-up 3 (2.6 years; n.390) in patients not treated (AChEI −/ME −), treated with acetylcholinesterase inhibitors only (AChEI +), memantine only (ME +), or both treatments (AChEI +/ME +)

Figure 2 illustrates the mean MMSE scores (± standard deviation) over time in the four treatment groups, assessed at baseline (time 0), time 1 (0.7 ± 0.3 years), time 2 (1.6 ± 0.5 years), and time 3 (2.6 ± 0.3 years). Over the follow-up period, changes in mean MMSE scores were as follows: − 1.7 points (SD: −1.5 to −1.9) in the untreated group (AChEI −/ME −; p for trend: 0.001), − 1.5 points (SD: −1.3 to −1.7) in the AChEI-only group (AChEI +; p for trend: 0.001), − 0.7 points (SD: −0.5 to −0.9) in the ME-only group (ME +; p for trend: 0.09), and + 0.7 points (SD: + 0.4 to + 1.0) in the combined treatment group (AChEI +/ME +; p for trend: 0.001).

**Fig. 3:**
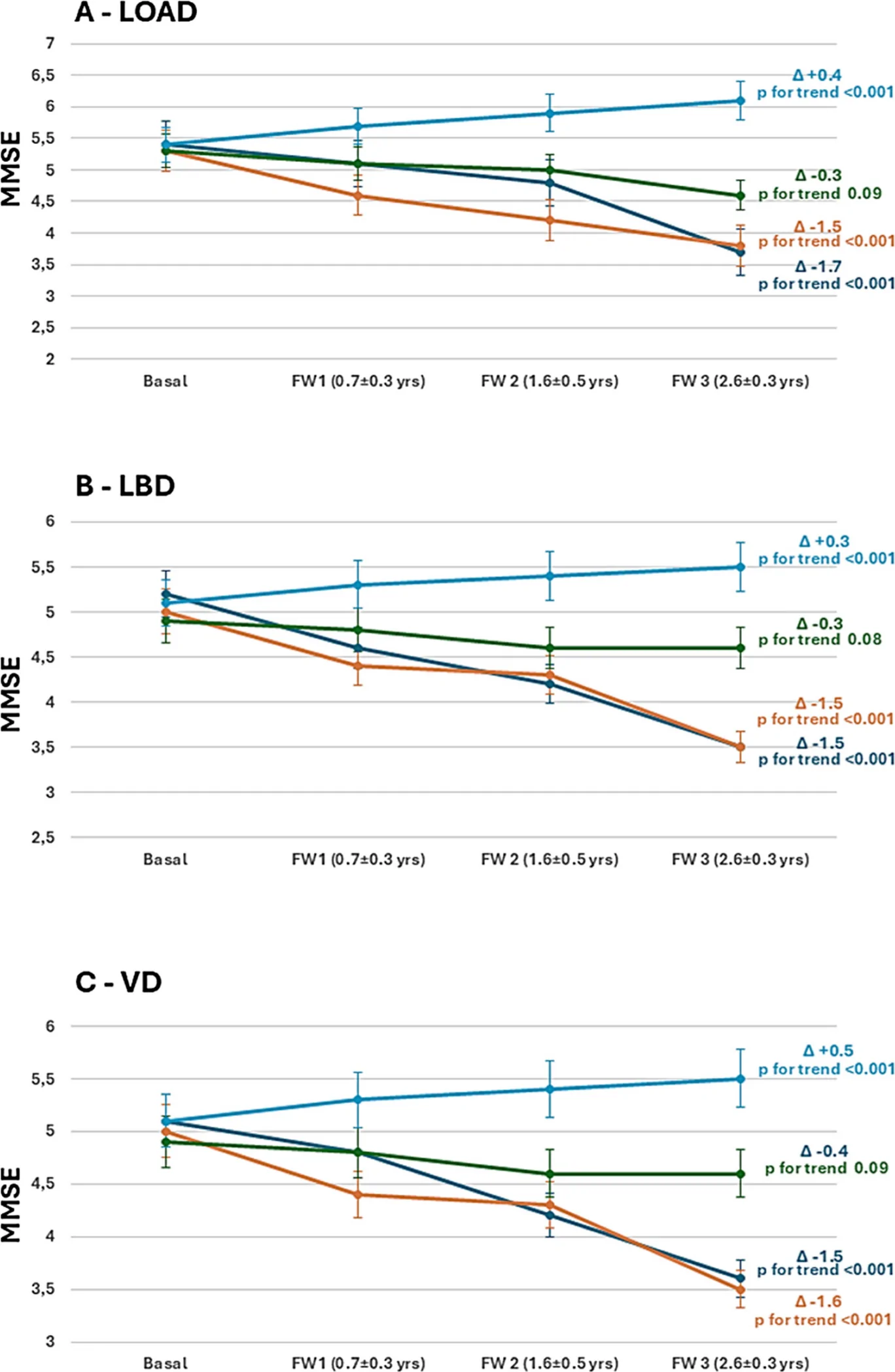
MMSE scores (mean ± SD) at baseline and at follow-up 1 (0.7 years), follow-up 2 (1.6 years), and follow-up 3 (2.6 years) in patients not treated (AChEI −/ME −), treated with acetylcholinesterase inhibitors only (AChEI +), memantine only (ME +), or both treatments (AChEI +/ME +), stratified by dementia type (LOAD: n.407; LBD: n.126; VD: n. 170)

Comparable trends were observed when MMSE trajectories were analyzed separately in patients affected by LOAD, LBD, and VD (Fig. 3). Among LOAD patients, changes in mean MMSE scores were as follows: − 1.5 points (SD: −1.1 to −1.9) in the AChEI −/ME − group (p for trend: 0.001), − 1.7 points (SD: −1.3 to −2.1) in the AChEI + group (p for trend: 0.001), − 0.3 points (SD: −0.05 to −0.55) in the ME + group (p for trend: 0.09), and + 0.4 points (SD: + 0.05 to + 0.75) in the AChEI +/ME + group (p for trend: 0.001). In patients with LBD, changes in mean MMSE scores were as follows: − 1.5 points (SD: −1.35 to −1.65) in the AChEI −/ME − group (p for trend: 0.001), − 1.5 points (SD: −1.35 to −1.65) in AChEI + group (p for trend: 0.001), − 0.3 points (SD: −0.1 to −0.5) in the ME + group (p for trend: 0.08), and + 0.3 points (SD: + 0.05 to + 0.55) in the AChEI +/ME + group (p for trend: 0.001). Finally, among patients with VD, changes in mean MMSE scores were as follows: − 1.6 points (SD: −1.4 to −1.8) in the AChEI −/ME − group (p for trend: 0.001), − 1.5 points (SD: −1.3 to −1.7) in the AChEI + group (p for trend: 0.001), − 0.4 points (SD: −0.15 to −1.65) in the ME + group (p for trend: 0.09), and + 0.5 points (SD: + 0.2 to + 0.8) in the AChEI +/ME + (p for trend: 0.001).

In summary, across all subtypes of dementia (LOAD, LBD, VD) we observed a significant worsening of the MMSE score in untreated patients as well as in those treated only with AChEI, a not-significant worsening of MMSE score in patients treated with ME alone, and a small although significant improvement of MMSE score in patients treated with ME plus AChEI. However, since LBD and VD subgroups were underpowered, these analyses should be considered as “exploratory”.

### Survival rates

**Fig. 4:**
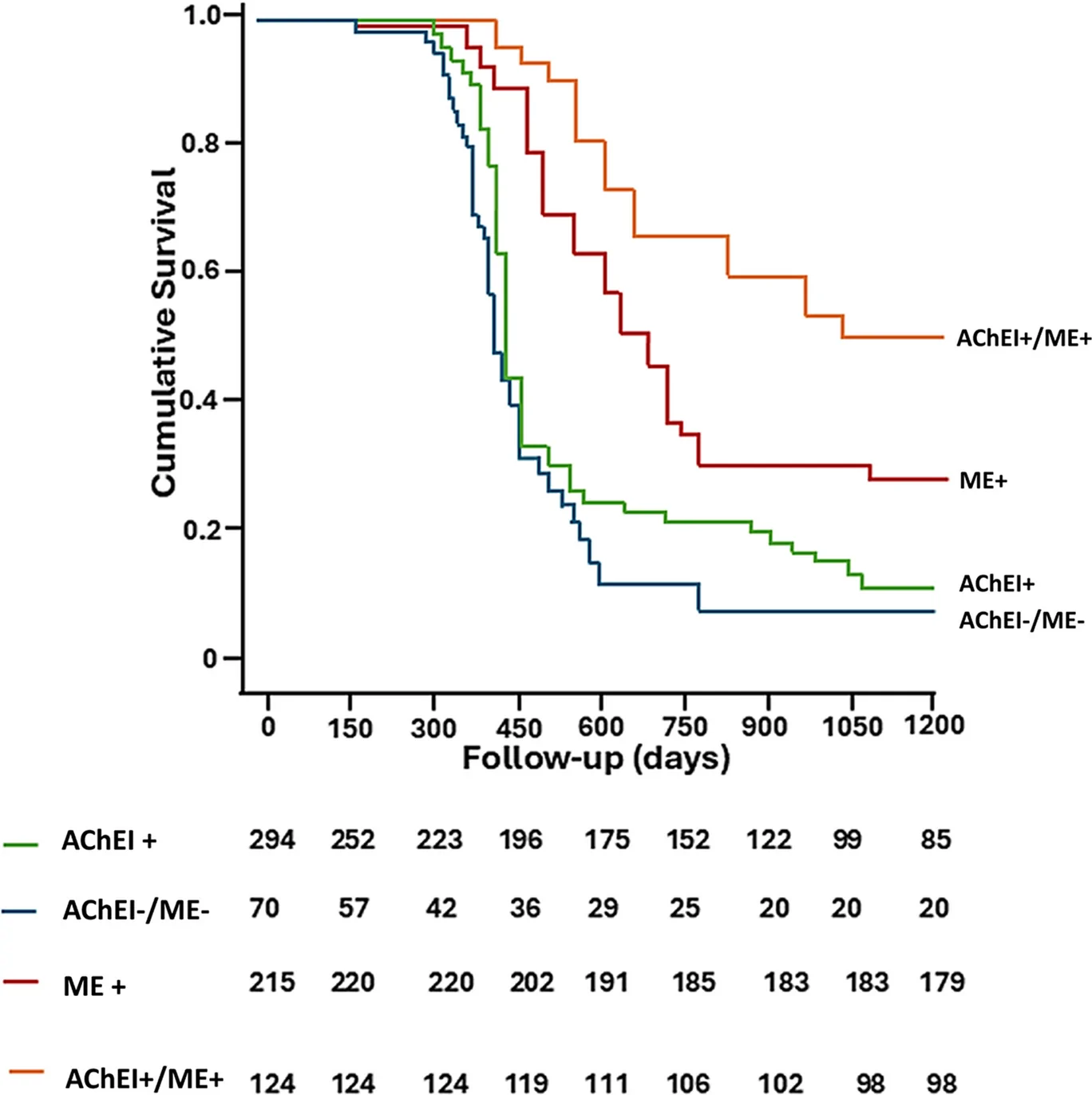
Kaplan–Meier survival curves for patients not treated (AChEI −/ME −; n. 70), treated with AChEI + (n. 294), ME + (n.215), or AChEI +/ME + (n.124) (Cox regression analysis; Log-Rank Mantel-Cox test: *p* = 0.001)

Figure 4 shows the Kaplan–Meier survival curves for the four treatment groups. At 2.6 years of follow-up, the estimated survival rates were 8% in the untreated group (AChEI −/ME −), 10% in the AChEI-only group (AChEI +), 28% in the ME-only group (ME +), and 49% in the combined treatment group (AChEI +/ME +), based on values calculated from the survival curves. Compared with untreated patients, AChEI monotherapy was not associated with a significant survival benefit (Log-Rank Mantel-Cox *p*:0.81). In contrast, both ME monotherapy and combination therapy with AChEI and ME were significantly associated with improved survival (Log-Rank Mantel-Cox *p*:0.001 for both), with the best outcomes in patients in the combination therapy group. Further comparisons revealed that patients treated with ME alone had significantly higher survival rates than those treated with AChEI alone, and that combined treatment with AChEI and ME conferred a survival advantage over either monotherapy (all Log-Rank Mantel-Cox *p*:0.001). Similar survival patterns were observed when the analysis was stratified by dementia subtype (data not shown).

In the Cox proportional hazards analysis (adjusted for age and sex) ME monotherapy was associated with a 22% reduction in all-cause mortality compared with no treatment (HR: 0.78; 95%CI: 0.65–0.94; *p*: 0.007). The combined AChEI +/ME + group exhibited the lowest mortality risk (HR: 0.61; 95%CI: 0.49–0.77; *p* < 0.001), while AChEI monotherapy showed no significant association (HR: 0.97; 95%CI: 0.80–1.18; *p*:0.75). When the Cox proportional hazards models were stratified by dementia subtype, consistent patterns were observed across all groups. In LOAD, both ME monotherapy (HR: 0.79; 95%CI: 0.63–0.98; *p*:0.03) and AChEI +/ME + combination therapy (HR: 0.59; 95%CI: 0.46–0.76; *p* < 0.001) were associated with significantly lower mortality compared with untreated patients, whereas AChEI monotherapy showed no effect (HR: 0.95; 95%CI: 0.76–1.19; *p*:0.65). In LBD, combination therapy AChEI +/ME + conferred the greatest survival benefit (HR: 0.57; 95%CI: 0.38–0.86; p:0.007), followed by ME monotherapy (HR: 0.75; 95%CI: 0.56–1.02; *p*:0.06). In VD, survival trends were directionally similar, but did not reach statistical significance (AChEI +/ME + : HR: 0.69; 95%CI: 0.46–1.03; *p*:0.07).

## Discussion

In the present analysis we investigated the potential long-term effects of AChEI and/or ME on cognitive function and mortality rates in older outpatients with different types of severe dementia, by using a real-world data from the NACC-UDS.

### Acetylcholinesterase inhibitors

In the present cohort, AChEI treatment was not associated with significant changes in MMSE scores or survival rates over a mean follow-up of 1.7 years (range:0.2–2.9 years). These findings align with the stances of several national drug regulatory agencies (e.g. Sweden, Netherlands, Italy, Austria, France, Belgium) which do not reimburse AChEI treatment in severe dementia due to uncertain clinical benefit. Previous evidence from both our group and other demonstrated that AChEI confer cognitive and survival advantages in patients with mild-to-moderate dementia [28], a finding corroborated by the meta-analysis by Truong et al. [5]. It is worth recalling that a few early randomized controlled trials (RCTs) from the 2000s reported short-term cognitive benefits of AChEI even in patients with severe AD [11–14]. However, several key methodological differences exist between those RCTs and our study. First, RCT participants were newly randomized and treatment-naïve, while our sample included patients already receiving AChEI for years; conversely, untreated patients may have discontinued AChEI due to intolerance or lack of efficacy. Second, the follow-up duration in RCTs was typically six months, whereas ours extended to over eighteen months. Third, our sample included patients with LBD and VD, not just AD; despite this broader diagnostic inclusion, no sub-group differences in MMSE trajectories or mortality were observed. Finally, the potential selection bias in our real-world cohort cannot be overlooked: patients continuing AChEI may have had a more aggressive disease course despite therapy, whereas untreated individuals might have experienced contraindications or milder trajectories. Therefore, in light of these considerations, it is plausible that the long-term effectiveness of AChEI diminishes as neurodegeneration advances, rendering residual cholinergic transmission insufficient to sustain clinical benefits.

### Memantine

Our results showed that ME therapy was associated with a stabilization of MMSE score over time (−0.7 points after 2.6 years; *p*:0.09) and a significant reduction in all-cause mortality of about 20% lower compared to untreated patients. The results of Cox regression analysis further support the survival advantage associated with ME and, especially, with AChEI + ME combination therapy. These findings are consistent with the Kaplan–Meier survival patterns and corroborate prior literature data showing reduced mortality among ME-treated patients. Our findings are consistent with RCT data showing ME efficacy in moderate-to-severe, but not mild, AD [16, 17, 29], as well as with the recent meta-analysis by Zolnowski-Kolp et al. [30], reporting a 19% mortality reduction associated with ME treatment. In contrast, the Literature evidence for ME in LBD is mixed. Some studies have found reduced mortality or behavioral improvements [18], while others failed to confirm these outcomes [31]; note that none of these studies specifically targeted patients with severe dementia. Thus, our findings may provide additional support for extending ME treatment to this population.

### AChEI and Memantine

In our study, the most favourable outcomes were observed in patients receiving both AChEI and ME. This group demonstrated a small but statistically significant improvement in MMSE score over time (+ 0.7 points; *p*:0.001), along with the greatest survival benefit (~ 40% reduction in mortality compared with untreated patients and those on AChEI alone; ~ 20% compared with ME monotherapy). Substantially, what we observed was a stabilization of cognitive functions and a reduction in total mortality in LOAD and LBD (with a similar but not significant trend in VD) when compared to untreated patients. However, this may have only a minimal effect on functional status and quality of life. In this regard, dedicated studies will be useful, incorporating non-cognitive endpoints such as independence in activities of daily living (ADL), behavioral symptoms, caregiver burden, and institutionalization risk.

Although the results from previous studies were heterogeneous, several of them support the potential of combination therapy. Tariot et al. reported cognitive and global functional benefits from the addition of ME to ongoing AChEI treatment in patients with moderate-severe AD over six months [20]. Similarly, Gareri et al. documented short-term MMSE improvements with combination therapy [22]. In contrast, Howard et al. [21] found no added benefit of ME when combined with AChEI over 52 weeks, while a meta-analysis by Farrimond et al. [32] described only a modest cognitive benefit. As for mortality, our results diverge somewhat from those of Lazzeroni et al. [23], who reported greater survival with ME monotherapy compared to both AChEI alone and combined therapy. However, a large retrospective French study by Havreng-Théry et al. [33] reported mortality reductions of 17%, 14%, and 26% for AChEI, ME, and the combination, respectively, with no significant differences between treatment groups, suggesting a potential, though not definitive, advantage for combination therapy.

The effect of ME on cognitive stabilization in moderate-severe dementia is biologically plausible. By modulating NMDA receptor-mediated excitotoxicity, ME protects against calcium overload and neuronal damage. Beyond the central nervous system, NMDA receptors are widely expressed in peripheral tissues; indeed, it has been shown that ME exerts a systemic anti-inflammation/anti-oxidative stress action [34], a cardio-protective effect by reduction in cardiac remodelling and fibrosis [35], and possibly anti-cancer effects by regulating tumour growth, invasion, and metastases in different cancer types [36]. All these mechanisms offer a potential mechanistic explanation for reduced overall mortality in patients treated with ME. As regards AChEI, the mechanisms proposed to underlie survival benefits in earlier disease stages include reduced cardiovascular risk, delayed frailty progression, improved neuropsychiatric symptoms (reducing the need for antipsychotics), and stabilization of cognitive functions [4, 5, 28]. However, in our sample including only patients with severe dementia, these AChEI benefits were not observed.

Anyhow, discontinuation of AChEI in advanced dementia remains a controversial decision. While some patients may benefit from deprescribing, due to adverse effects or futility, others may experience clinical deterioration after withdrawal. In the study by Howard et al. [21], continued AChEI therapy was associated with a mean MMSE advantage of 1.9 points over 12 months. Rebound cognitive or behavioural worsening has also been reported [10]; therefore, deprescribing decisions must be individualized. Predictors of AChEI discontinuation in nursing home settings include poor prognosis, weight loss, communication difficulties, recent hospitalization, and hospice admission [37, 38]. Of note, only 9.9% of our sample were not receiving AChEI or ME, indicating limited deprescribing practices in this real-world setting.

### Limitations of the study

Our study has several limitations. First, the sample size was modest due to stringent inclusion criteria; moreover, only a low proportion of patients were not receiving any dementia medication.

Second, data on functional status (e.g. ADL, Clinical Dementia Rating Scale—CDR) and comorbidities were incomplete and could not be reliably analyzed. It should be acknowledged that untreated patients might represent a frailer subgroup (e.g. more advanced disease, more disability, greater comorbidity burden, limited caregiver support; all of which could have influenced both treatment assignment and outcomes). In our baseline comparisons, no statistically significant differences were observed among the treatment groups with respect to principal comorbidities or concomitant therapies; therefore, Propensity Score Matching or Inverse Probability of Treatment Weighting was not performed. However, despite adjustment for age and sex, residual confounding cannot be excluded.

Third, cognitive assessment relied on the MMSE, which may lack sensitivity to detect subtle or domain-specific changes, and is susceptible to measurement variability [39]; thus, small but clinically meaningful differences in cognition may have been missed.

Fourth, the specific causes of death were not available, and this does not allow further speculations about the potential protective effect of these drugs.

## Conclusions

In this real-world cohort of older outpatients with severe dementia from the NACC-UDS, treatment with AChEI did not significantly influence cognitive decline or overall mortality over a mean 1.7 years follow-up. In contrast, ME seemed to be associated with a stabilization of cognitive function and a significant reduction in total mortality, but the strongest cognitive effect and survival benefit was demonstrated for the combination therapy (ME + AChEI). Despite the limitations inherent in an observational study and the possibility of unmeasured confounding factors, our findings may contribute to clinical decision-making regarding the continuation or discontinuation of these dementia therapies in patients with advanced disease.

## Supplementary Information

Below is the link to the electronic supplementary material.

ESM1 (DOCX 27.8 KB)
