# Supplementary material for: Effectiveness of acetylcholinesterase inhibitors and memantine in patients affected by severe dementia
Source: GeroScience. 2025 Dec 15;48(5):7191–202. doi: 10.1007/s11357-025-02052-3 (PMC13601481; doi:10.1007/s11357-025-02052-3)
Supplement: Supplementary file 1 [file 11357_2025_2052_MOESM1_ESM.docx]

**Supplementary Table 1**: Principal comorbidities and drug treatments in the sample according to the treatment with AChEI/ME.

|  | **AChEI-/ME-**  **N=70** | **AChEI +**  **N=294** | **ME +**  **N=215** | **AChEI+/ME+**  **N=124** | **P** |
| --- | --- | --- | --- | --- | --- |
| **COMORDIBITIES** | | | | | |
| **Ischemic Heart Disease** | 25 (35.7) | 91 (30.9) | 75 (34.8) | 42 (33.8) | 0.39 |
| **Atrial Fibrillation** | 20 (28.5) | 81 (27.5) | 63 (29.3) | 36 (29.0) | 0.92 |
| **Heart Failure** | 28 (40.0) | 102 (34.6) | 79 (36.7) | 47 (37.9) | 0.88 |
| **Cerebrovascular disease** | 18 (25.2) | 53 (18.0) | 38 (17.6) | 28 (22.5) | 0.82 |
| **Hypertension** | 34 (48.5) | 150 (51.0) | 108 (50.2) | 61 (49.1) | 0.93 |
| **Diabetes** | 8 (11.4) | 43 (14.6) | 31 (14.1) | 19 (15.3) | 0.78 |
| **Hypercholesterolemia** | 15 (21.4) | 57 (19.3) | 46 (21.3) | 25 (20.1) | 0.73 |
| **Urinary Incontinence** | 13 (18.5) | 48 (16.3) | 39 (18.1) | 20 (16.1) | 0.65 |
| **Depression (last 2 years)** | 26 (37.1) | 127 (43.1) | 81 (37.6) | 45 (36.2) | 0.63 |
| **Extrapyramidal syndrome** | 12 (17.1) | 44 (14.9) | 33 (15.3) | 21 (16.9) | 0.75 |
| **DRUGS** |  |  |  |  |  |
| **Anti-Adrenergic agents** | 11 (15.7) | 36 (12.4) | 30 (13.9) | 17 (13.7) | 0.79 |
| **Antihypertensive drugs** | 31 (44.2) | 146 (49.6) | 101 (46.9) | 57 (45.9) | 0.63 |
| **Lipid-lowering drugs** | 13 (18.5) | 50 (17.0) | 33 (15.3) | 20 (16.1) | 0.83 |
| **Antidiabetic agents** | 6 (8.5) | 36 (12.2) | 26 (12.0) | 13 (10.4) | 0.76 |
| **Antipsychotic agents** | 6 (8.5) | 32 (10.8) | 22 (10.2) | 14 (11.2) | 0.88 |
| **Antidepressants** | 21 (30.0) | 119 (40.4) | 85 (39.5) | 45 (36.2) | 0.73 |
| **Anxiolytic agents** | 21 (30.0) | 57 (19.3) | 625 (30.2) | 39 (31.4) | 0.82 |

AChEI: acetylcholinesterase inhibitors; ME: memantine; LOAD: late onset Alzheimer’s disease; LBD: Lewy body disease; VD: vascular dementia
